# Interaction of the Fungal Metabolite Harzianic Acid with Rare-Earth Cations (La^3+^, Nd^3+^, Sm^3+^, Gd^3+^)

**DOI:** 10.3390/molecules27061959

**Published:** 2022-03-17

**Authors:** Gaetano De Tommaso, Maria Michela Salvatore, Antonietta Siciliano, Alessia Staropoli, Francesco Vinale, Rosario Nicoletti, Marina DellaGreca, Marco Guida, Francesco Salvatore, Mauro Iuliano, Anna Andolfi

**Affiliations:** 1 Department of Chemical Sciences, University of Naples Federico II, 80126 Naples, Italy; gaetano.detommaso@unina.it (G.D.T.); mariamichela.salvatore@unina.it (M.M.S.); dellagre@unina.it (M.D.); 2 Institute for Sustainable Plant Protection, National Research Council, 80055 Portici, Italy; alessia.staropoli@unina.it (A.S.); frvinale@unina.it (F.V.); 3 Department of Biology, University of Naples Federico II, 80126 Naples, Italy; antonietta.siciliano@unina.it (A.S.); marco.guida@unina.it (M.G.); 4 Department of Agricultural Sciences, University of Naples Federico II, 80055 Portici, Italy; rosario.nicoletti@crea.gov.it; 5 Department of Veterinary Medicine and Animal Productions, University of Naples Federico II, 80137 Naples, Italy; 6 BAT Center—Interuniversity Center for Studies on Bioinspired Agro-Environmental Technology, University of Naples Federico II, 80055 Portici, Italy; 7 Council for Agricultural Research and Economics, Research Centre for Olive, Fruit and Citrus Crops, 81100 Caserta, Italy

**Keywords:** complex formation, *Trichoderma*, fungal metabolites, tetramic acid, rare-earth elements

## Abstract

Rare-earth elements are emerging contaminants of soil and water bodies which destiny in the environment and effects on organisms is modulated by their interactions with natural ligands produced by bacteria, fungi and plants. Within this framework, coordination by harzianic acid (H_2_L), a *Trichoderma* secondary metabolite, of a selection of tripositive rare-earth cations Ln^3+^ (Ln^3+^ = La^3+^, Nd^3+^, Sm^3+,^ and Gd^3+^) was investigated at 25 °C, and in a CH_3_OH/0.1 M NaClO_4_ (50/50 *w/w*) solvent, using mass spectrometry, circular dichroism, UV–Vis spectrophotometry, and pH measurements. Experimental data can be satisfactorily explained by assuming, for all investigated cations, the formation of a mono-complex (LnL^+^) and a bis-complex (LnL_2_^−^). Differences were found between the formation constants of complexes of different Ln^3+^ cations, which can be correlated with ionic radius. Since gadolinium is the element that raises the most concern among lanthanide elements, its effects on organisms at different levels of biological organization were explored, in the presence and absence of harzianic acid. Results of ecotoxicological tests suggest that harzianic acid can decrease gadolinium biotoxicity, presumably because of complex formation with Gd^3+^.

## 1. Introduction

Fungi are particularly prolific sources of secondary metabolites, which are low molecular weight compounds fulfilling very different functions (e.g., mediating communication and interactions with organisms and facilitating nutrient acquisition) and multiple bioactivities (e.g., antibacterial, antioxidant, antiproliferative). The staggering variation in the chemical structure of these compounds explains their broad range of activities and functions [1,2,3,4,5,6,7,8]. In fact, there is currently an increasing interest in secondary metabolites for discovering new lead compounds that may perform useful functions for humans. 

Harzianic acid is a fungal secondary metabolite, belonging to the subgroup of dienoyl tetramic acids [9], essentially produced by fungi from the genus *Trichoderma* [10,11]. It has raised the interest of several researchers because of its valuable bioactivities, including plant growth promotion, as well as antimicrobial and antibiofilm activities [12,13,14,15].

Recently, it was reported that harzianic acid has the capacity to form stable complexes with an assortment of metals (e.g., Cu, Fe, Ni, Pb, Zn), some of which are useful bio-elements, while others are well-known toxic heavy metals, which can contaminate soil and surface and ground waters [16,17]. 

On the other side, contamination of soil and waters by rare-earth elements is an emerging issue because these elements and their compounds are vital to the production of an increasing number of technological products employed for energy production and storage, electronics, and military applications [18,19,20,21]. Several studies have reported the effects of lanthanides on microorganisms and plants that can interact with rare-earth elements through the great diversity of organic compounds that they are able to produce [22,23,24]. For instance, species of *Trichoderma* showed good tolerance to the presence of rare-earth elements in both solid and liquid media, and it is supposed that these effects could be related to the production of specific compounds able to detoxify the substrate [25]. Organic substances are crucial for the potential phenomena of adsorption or chelation, which may influence the impact of these metals on organisms and microorganisms sharing the same ecological niche. Thus, the last decade has seen an increasing interest among the scientific community for the coordination properties of organic ligands toward rare-earth elements [26,27,28,29]. 

Among rare-earth elements, the anthropogenic input of gadolinium in soil and waters raises the most concern because of its widespread use in medical diagnostics as a contrast agent [30,31,32,33]. For this purpose, a number of molecules to be used as ligands of gadolinium have been synthetized in order to satisfy specific medical requirements [30,34,35]. It is not surprising that levels of gadolinium in water bodies are frequently observed to reach ng L^−1^ levels and are predicted to increase in the future [36]. Anomalous and alarming levels of lanthanum and samarium in the environment are also observed because of their use in solid oxide fuel cells, as catalysts, and as additives to improve glass properties [36,37,38].

Since the destiny of metals in the environment and their effects on organisms and biological systems ultimately depend on their chemical properties, and eminently on their interactions with natural ligands, we have engaged ourselves in the study of complex formation equilibria between a selection of tripositive rare-earths cations (Ln^3+^) and the secondary metabolite harzianic acid. Obviously, our selection of lanthanides includes gadolinium, lanthanum, and samarium, which are used in industries, and their anthropogenic input into the environment is predicted to increase in the next few decades. We also included neodymium in our investigation to fill the gap between lanthanum and samarium, which may be useful to discover trends existing in the lanthanide series of elements. 

In this paper, we summarize our findings on the coordination of lanthanum, neodymium, samarium, and gadolinium tripositive cations by harzianic acid. We employed HPLC–ESI–HRMS experiments, circular dichroism (CD), and UV–Vis spectrophotometric absorption data, as well as accurate pH measurements, to characterize bonding interactions between harzianic acid and the selected lanthanides. Due to the low solubility of harzianic acid in water, the stoichiometry and complex formation constants of selected lanthanides cations with harzianic acid were evaluated in a CH_3_OH/0.1 M NaClO_4_ (50/50 *w/w*) solvent.

Finally, in order to reveal possible changes in toxicity of rare-earth cations caused by the complexation with harzianic acid, we employed ecotoxicological tests to explore gadolinium effects on organisms at different levels of biological organization (i.e., on Gram-negative bacterium *Aliivibrio fischeri,* green alga *Raphidocelis subcapitata,* and microcrustacean *Daphnia magna*) in the absence and in presence of harzianic acid. 

## 2. Results and Discussion

### 2.1. Harzianic Acid 

Enantiomerically pure (*S,S)*-harzianic acid employed in this study was extracted from cultures of the strain L1 of *Trichoderma pleuroticola* recovered from the gastropod *Melarhaphe neritoides* as described in a previous study [17]. The isolated compound showed the same NMR spectroscopic characteristics as those of an authentic standard compound [39]. Due to its low solubility in water, all equilibria involving harzianic acid were studied in a CH_3_OH/0.1 M NaClO_4_ (50/50 *w*/*w*) solvent.

**Figure 1:**
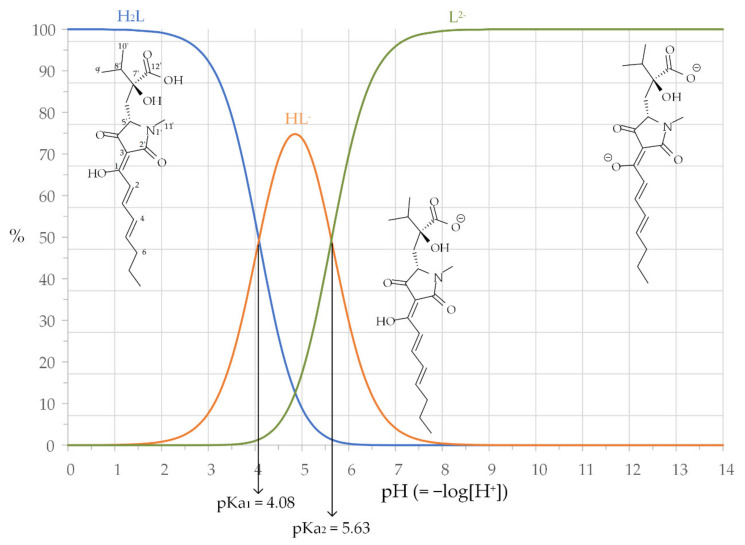
Distribution diagram of harzianic acid (H_2_L). Reported dissociation constants of harzianic acid were separately determined at 25 °C in the mixed solvent CH_3_OH/0.1 M NaClO_4_ (50:50 *w/w*) [16]. Inserts: structures of harzianic acid (H_2_L) and its acid-dissociated products (HL^−^ and L^2−^).

As shown in the distribution diagram and structures in Figure 1, harzianic acid is a diprotic acid, which is indicated as H_2_L in subsequent sections. Dissociation of the acidic protons from H_2_L readily occurs so that the full deprotonated species (L^2−^) prevails over the diprotonated (H_2_L) and monoprotonated (HL^−^) species at the prevailing pH of biological and natural systems. This is a crucial factor that contributes to the success of harzianic acid as a complexing agent for metal cations in biological and natural environments because protonation of the binding sites of ligands which are weak acids is a collateral reaction that may prevent bonding to metal cations. 

### 2.2. Coordination Properties of Harzianic Acid toward La^3+^, Nd^3+^, Sm^3+^ and Gd^3+^

The complexing properties of harzianic acid toward four tripositive rare-earth cations were studied by using mass spectrometry and potentiometric and spectrophotometric techniques. Tripositive rare-earth cations considered in this study (i.e., La^3+^, Nd^3+^, Sm^3+^, Gd^3+^) are indicated with the general symbol Ln^3+^ in subsequent sections. 

First, in order to obtain an indication of interactions occurring between rare-earth elements and harzianic acid, solutions of Ln^3+^ cations and harzianic acid were submitted for HPLC–ESI–HRMS analysis. High-resolution mass spectra were acquired on solutions prepared by mixing 500 µL of 2 mM solution of each Ln^3+^ cation and 500 µL of harzianic acid 1 mg mL^−1^ in methanol. 

The most abundant ions in the mass spectrum (MS) of each solution are reported in Table 1. In all mass spectra, peaks corresponding to adducts of harzianic acid with hydrogen and sodium were detected, i.e., [H_2_L + H]^+^ (*m*/*z* 366.1921) and [H_2_L + Na]^+^ (*m/z* 388.1730). Furthermore, mass peaks attributable to ions that contain both Ln^3+^ cations and harzianic acid, normally in a 1:2 metal-to-ligand ratio, were also observed. 

Further evidence for bonding interactions occurring between Ln^3+^ cations and harzianic acid was gathered by employing circular dichroism (CD) and UV–Vis spectroscopy. 

To this end, CD and UV–Vis spectra were acquired, at 25 °C, on solutions which analytical composition was accurately known by preparation, where free proton concentration, [H^+^], was measured with a pH indicator glass electrode calibrated to respond to free proton concentration in the CH_3_OH/0.1 M NaClO_4_ (50/50 *w/w*) solvent. Thus, in the following sections, the standard symbol pH merely indicates the anti-logarithm of the molar concentration of solvated protons in the tested solutions (i.e., we define pH = −log[H^+^]).

As shown by the analytical array (1), for the way the test solutions (TSs) to be presented to the CD and UV-Vis spectrometers were prepared, we used four analytical variables (i.e., $C_{\mathrm{Ln}}$ = total molar concentration of Ln^3+^ cation; $C_{H_{2}L}$ = total harzianic acid molar concentration; $C_{H}$ = molar analytical excess of strong acid; $C_{\mathrm{OH}}$ = molar analytical excess of strong base) to specify their analytical composition:

(1) $${\left\{C_{\mathrm{Ln}}\;M\;\mathrm{Ln}{\left({\mathrm{ClO}}_{4}\right)}_{3}+C_{H_{2}L}{\;M\;H}_{2}L+C_{H}{\;M\;\mathrm{HClO}}_{4}+C_{\mathrm{OH}}^{\;}M\;\mathrm{NaOH},\;\;\;\;\;\;\left[H^{+}\right]=h\right\}}^{\mathrm{TS}}$$

For each Ln^3+^ cation, CD and UV–Vis spectra were acquired on test solutions of different analytical compositions in which, as a broad indication, $C_{\mathrm{Ln}}$ and $C_{H_{2}L}$ achieved values between ≈10^−4^ M and ≈10^−5^ M and pH ranged from ≈2.5 to ≈9. 

**Figure 2:**
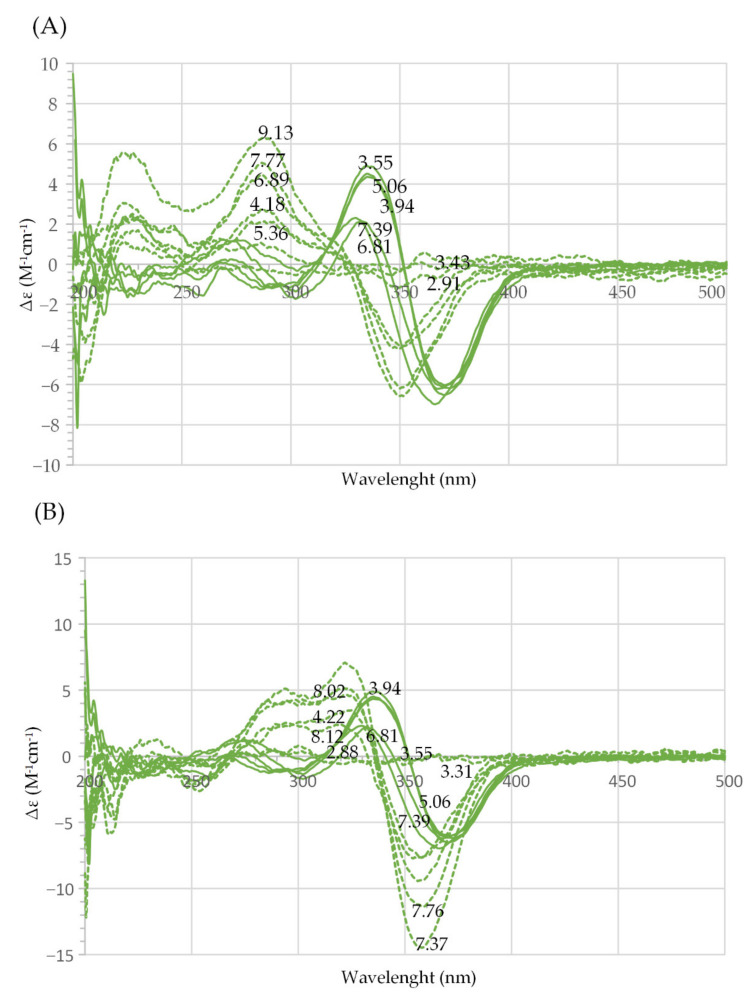
(**A**) Superimposition of far-UV circular dichroism (CD) spectra of harzianic acid (≤1.72 × 10^−4^ M, dashed curves) and of Gd^3+^–harzianic acid system at 1:1 ligand-to-metal ratio (continuous curves) in CH_3_OH/0.1 M NaClO_4_ (50:50 *w*/*w*) at different pH values; (**B**) superimposition of far-UV circular dichroism (CD) spectra of Gd^3+^–harzianic acid system (full lines) and La^3+^–harzianic acid system (dashed lines) at 1:1 ligand-to-metal ratio in CH_3_OH/0.1 M NaClO_4_ (50:50 *w*/*w*) at different pH values: $C_{\mathrm{Gd}}$ ≤ 6.44 × 10^−5^ M, $C_{H_{2}L}/C_{\mathrm{Gd}}$ = 1.00; $C_{\mathrm{La}}$ ≤ 3.82 × 10^−5^ M, $C_{H_{2}L}/C_{\mathrm{La}}$ = 0.995. In (**A**,**B**), numerical labels on curves indicate the corresponding pH.

As shown in Figure 2A, CD spectra of solutions of harzianic acid (dashed curves) revealed a negative Cotton effect, with a positive peak at about 280 nm and a negative peak at about 350 nm. As it could be easily predicted, the CD signal showed a strong dependence on pH, which governs the equilibrium concentrations of H_2_L, HL^−^ and L^2^^−^.

Similar patterns were observed for CD spectra of solutions containing harzianic acid and Ln^3+^ cations, but the CD peaks were observed at longer wavelengths, as exemplified in Figure 2A for Gd^3+^ (continuous curves). Furthermore, CD signals were distinctly different for different Ln^3+^ cations, as can be deduced from Figure 2B and from Supplementary Figures S1–S5. 

These facts strengthen the evidence produced by HPLC–ESI–HRMS data for bonding interactions occurring in the investigated solutions between Ln^3+^ cations and harzianic acid.

However, a part from that, ultimately, the stoichiometry and formation constants, at 25 °C and in a CH_3_OH/0.1 M NaClO_4_ (50/50 *w*/*w*) solvent, of complexes formed by Ln^3+^ cations with harzianic acid have been evaluated from the UV-Vis absorption data which are amenable to quantitative analysis by the Hyperquad program suite [40,41].

**Figure 3:**
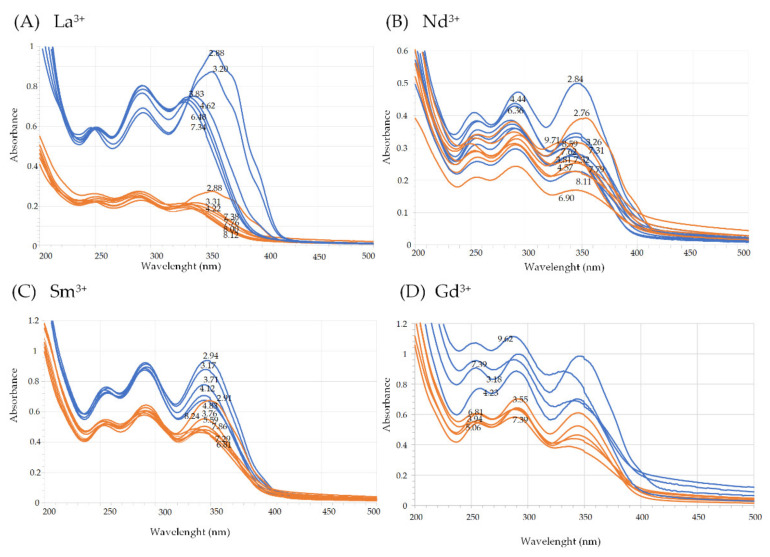
Raw UV–Vis spectra of (**A**) solutions of La^3+^ and harzianic acid, (**B**) solutions of Nd^3+^ and harzianic acid, (**C**) solutions of Sm^3+^ and harzianic acid, and (**D**) solutions of Gd^3+^ and harzianic acid with accurately known analytical composition and pH (=−log[H^+^]). For each metal cation, absorption spectra define two sets differentiated by color: orange-colored spectra were acquired on solutions with nearly equal concentration of the ligand and metal; blue spectra were acquired on solutions with concentration of the ligand nearly twice that of the metal cation. Numerical labels on curves indicate the corresponding pH. Details on ligand and metal concentrations employed to collect the UV–Vis spectra are reported in Table 2 for convenience.

For each Ln^3+^ cation, from the measured UV-Vis spectra a data matrix (Abs matrix) can be created based on the absorbances (*A_ik_*) measured at the wavelength specified by the row label for a test solution which analytical composition is specified by the column label. Thus, a column of the Abs matrix contains the UV-Vis spectrum of one test solution. UV–Vis spectra in the four Abs matrices (one matrix for each investigated rare-earth element), collected in this work and on which all successive evaluations were based, are visually presented in Figure 3. 

Although each solution in Figure 3 had its own analytical composition, the acquisition workflow was planned so that, for each Ln^3+^ cation, the full collection of absorption spectra (i.e., columns of the Abs matrix) could be grouped into two sets. Spectra in each set were acquired on test solutions with identical ligand-to-metal concentration ratio (i.e., $C_{H_{2}L}/C_{\mathrm{Ln}}$ = *constant*). In particular, spectra in the first set had a ligand-to-metal concentration ratio very close to 1, and spectra in the second set had a ligand-to-metal concentration ratio very close to 2. For instance, orange-colored spectra in Figure 3A were acquired on La^3+^–harzianic acid solutions in which $C_{H_{2}L}/C_{\mathrm{La}}$ = 1.00, while blue-colored spectra were acquired on solutions with $C_{H_{2}L}/C_{\mathrm{La}}$ = 2.04. However, spectra in each set had different values of $C_{H_{2}L}$, $C_{\mathrm{Ln}}$ and pH (as indicated by numerical labels on curves in Figure 3). This acquisition strategy is useful because it can help in making inferences from visual inspection of data. For instance, from Figure 3, a clear separation is observed between spectra in the first set ($C_{H_{2}L}/C_{\mathrm{Ln}}≅1)$ and spectra in the second set $(C_{H_{2}L}/C_{\mathrm{Ln}}≅2),$ which may be interpreted as an omen of the formation of complex species in the corresponding solutions. Table 2 exposes the details of the analytical compositions of solutions employed to acquire data in Figure 3.

Furthermore, in order to define the stoichiometry and formation constants of chemical species responsible for data in Figure 3, the Abs matrices were presented, one by one, to the Hyperquad program, which evaluates an assumed speciation model by a process of data simulation. 

A speciation model is defined with reference to general reaction indicated in (2) from which any chemical species present in the investigated solutions is supposed to be formed.

(2) $$p{\mathrm{Ln}}^{3+}+qL^{2-}+rH^{+}⇋{\mathrm{Ln}}_{p}L_{q}H_{r}^{\left(3p-2q+r\right)+}\;\;\;\;\;\beta_{pqr}=\frac{\left[{\mathrm{Ln}}_{p}L_{q}H_{r}^{\left(3p-2q+r\right)+}\right]}{{\left[{\mathrm{Ln}}^{3+}\right]}^{p}[L^{2-}{]}^{q}[H^{+}{]}^{r}}\;\;\;\;\;\;\;\;\;$$

Thus, any chemical species in the investigated solutions is defined by the array of stoichiometric coefficients (*p q r*), and a model is a collection of (*p q r*) arrays that define the species that are supposed to be present in the tested solutions. Beyond the general complex species ${\mathrm{Ln}}_{p}L_{q}H_{r}^{\left(3p-2q+r\right)+}$, the model can accommodate ligand protonation reactions (*p* = 0, *q* = 1, *r* > 0) and hydroxy complexes of Ln^3+^ (*p* > 0, *q* = 0, *r* < 0). For instance, fully protonated harzianic acid corresponds to species (0, 1, 2), while the hydroxy complex Ln(OH)^2+^ corresponds to species (1, 0, −1) since it is supposed to be formed through the reaction: Ln^3+^ + H_2_O – H^+^ ⇋ Ln(OH)^2+^.

Finally, the model to be evaluated and the pertinent Abs matrix were presented to Hyperquad which algorithm has the logic for finding the values of the $\beta_{pqr}$ equilibrium constants best reproducing the experimental matrix by minimizing the sum of squared residuals, U, as defined by Equation (3).

(3) $$U=\sum_{i}\sum_{k}{\left(A_{ik}-A_{ik}^{c}\right)}^{2}$$

In Equation (3), $A_{ik}$ represents the measured absorbance in row *i* and column *k* of the Abs matrix, and $A_{ik}^{c}$ is the corresponding absorbance calculated on the basis of the assumed model. Once a minimum value of U is found, Hyperquad outputs the $\beta_{pqr}$ values that produce, for the evaluated model, the best fit of the experimental data and the corresponding estimated standard deviations.

Determination of formation constants by this process is a demanding task since, in general, several models have to be evaluated and compared in order to select the one that most closely reproduces the experimental data. Furthermore, overfitting the data with models, which postulate a number of species greater than justified by experimental errors, is a possible degeneration of the minimization process that must be avoided and against which the program provides no safeguards. In order to decrease the risk of creating a chemically meaningless model by overfitting the experimental data, the ionic product of water *K*_w_ = 10^−14.5^ [42] and the ligand protonation constants $\beta_{011}$ = 10^5.63^ and $\beta_{012}$ = 10^9.71^, which can be calculated from the acid dissociation constants of harzianic acid determined separately in the CH_3_OH/0.1 M NaClO_4_ (50/50 *w/w*) solvent and are reported in Figure 1, were kept unchanged during the minimization process. For the same reason, in the evaluated models, only the hydroxy complex Ln(OH)^2+^ which, for the investigated lanthanides, is the prevailing species in water over an extended pH and range of metal concentrations, was included [43]. 

When this recursive procedure was implemented on our Abs matrices, we found that, for all Ln^3+^ cations, the very simple model consisting of the already known species LH^−^ (*p* = 0, *q* = 1, *r* = 1), LH_2_ (*p* = 0, *q* = 1, *r* = 2), Ln(OH)^2+^ (*p* = 1, *q* = 0, *r* = −1), and of the new complex species LnL^+^ (*p* = 1, *q* = 1, *r* = 0) and LnL^2−^ (*p* = 1, *q* = 2, *r* = 0) was the most parsimonious model that reproduces the UV–Vis data within experimental errors. 

The $\beta_{110}$ (shortly $\beta_{11}$) and $\beta_{120}$ (shortly $\beta_{12}$) formation constants of the LnL^+^ and LnL_2_^−^ complexes of Ln^3+^ cations investigated in this study are presented in Table 3.

**Figure 4:**
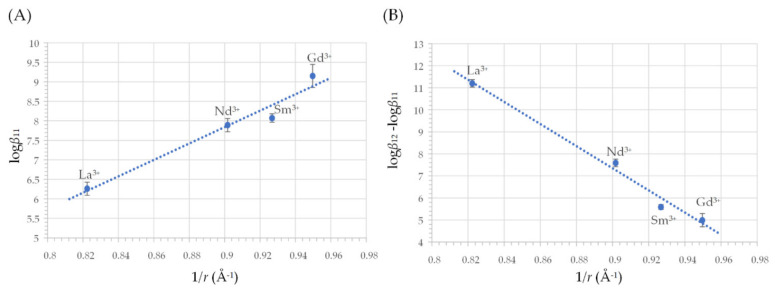
Correlation of formation constants from Table 3 with the radius (i.e., *r*) of Ln^3+^ cations: La^3+^ (*r* = 1.216 Å), Nd^3+^ (*r* = 1.109 Å), Sm^3+^ (*r* = 1.079 Å), Gd^3+^ (*r* = 1.053 Å) [43]: (**A**) equilibrium constant (*β*_11_) for reaction Ln^3+^ + L^2−^ ⇋ LnL^+^; (**B**) equilibrium constant (*K*_12_ = *β*_12_/*β*_11_) for reaction LnL^+^ + L^2−^ ⇋ LnL_2_^−^. L^2−^= (harzianic acid)^2−^. Dotted lines are least squares regression lines, and error bars represent three times the estimated standard deviation (3*σ*).

In Figure 4, the logarithm of formation constants of LnL^+^ complexes (log*β*_11_) and of the stepwise formation constants log*K*_12_ = log (*β*_12_/*β*_11_), of LnL_2_^−^ complexes from LnL^+^, is plotted as functions of the reciprocal of the radius (1/*r*) of Ln^3+^ cations. As expected, *β*_11_ increases as the ionic radius decreases. However, an opposite and remarkably unusual trend is observed for *K*_12_, which increases with ionic radius. In both cases, an exceptionally good linear correlation of log*β*_12_ and log*K*_12_ with 1/*r* is observed.

**Figure 5:**
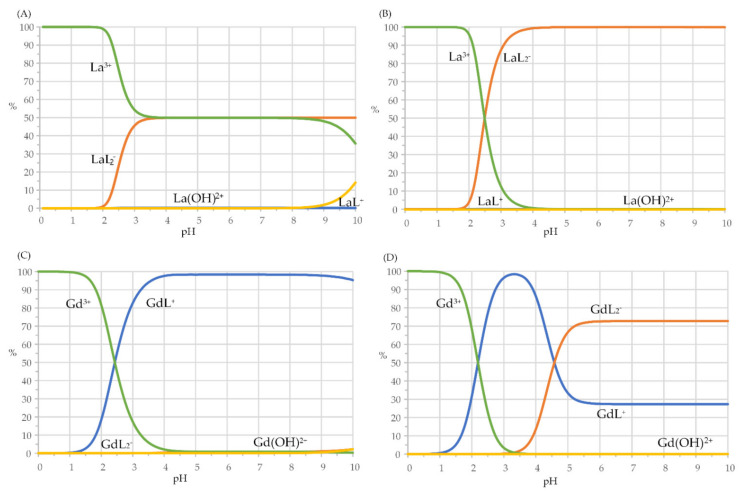
Distribution diagrams for: La^3+^–harzianic acid and Gd^3+^–harzianic acid systems: (**A**,**C**) equal total concentrations (10^−4^ M) of harzianic acid and metal cations; (**B**,**D**) harzianic acid total concentration (2 × 10^−4^ M) twice the total concentration of metal cations.

In order to expose the chemistry which, as a consequence of the formation constants in Table 3, takes place in solutions of harzianic acid and Ln^3+^ cations, in Figure 5 are presented distribution (or speciation) diagrams of La^3+^ (which has the lowest value of *β*_11_ and the largest value of *K*_12_) and of Gd^3+^ (which, on the contrary, has the largest value of *β*_11_ and the smallest value of *K*_12_) in solutions with a ligand to metal ratio equal 1 and 2.

As can be seen from Figure 5, starting from pH ≅ 2.5, while GdL^+^ is the prevailing species in solutions with a ligand-to-metal ratio equal to 1, and species GdL_2_^−^ prevails in solutions with a ligand-to-metal ratio equal to 2 at neutral and alkaline pH, in the case of La^3+^, species LaL^+^ is always a minor species, and the bis-complex (LaL_2_^−^) appears to be formed in a single step directly from the La^3+^ cation. Under analogous conditions, speciation of neodymium and samarium follows patterns more similar to the speciation of gadolinium than those of lanthanum since NdL^+^ and SmL^+^ mono-complexes predominate on wide pH ranges in solutions with equal concentrations of the ligand and metal, and the bis-complexes are the prevailing species in solutions with ligand-to-metal ratio equal to 2. 

Another way in which differences in the capability of harzianic acid to complex the different tripositive lanthanides cations can be explored is by using the conventional parameter “pM”. For a defined metal–ligand couple, pM represents the anti-logarithm of the free concentration of the metal cation (i.e., −log[Ln^3+^] in the present case) in a reference solution of pH = 7.4, where the total concentrations of the metal cation and of the ligand are, respectively 1.0 × 10^−6^ M and 1.0 × 10^−3^ M. By consequence, a larger pM value corresponds to a lower concentration of the free metal ion in solution at equilibrium and, in abstract, to a higher capability of the ligand to complex the metal cation. pM can be used to compare the binding ability of different ligands toward a given metal cation or, vice versa, to reveal differences in affinity of a given ligand for different cations irrespective of the stoichiometry of the formed complexes [44].

**Figure 6:**
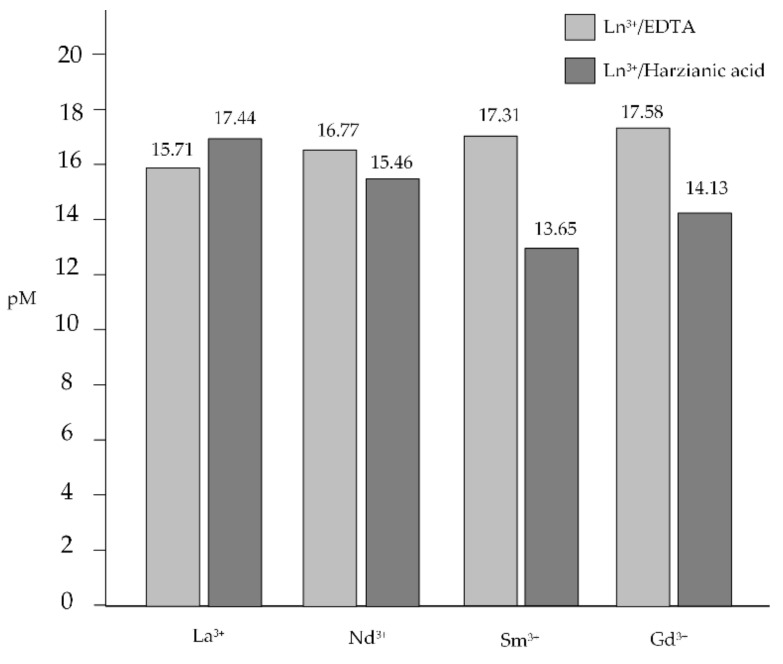
pM values calculated for La^3+^–harzianic acid, Nd^3+^–harzianic acid, Sm^3+^–harzianic acid, and Gd^3+^–harzianic acid systems using equilibrium constants determined in this paper in the CH_3_OH/0.1 M NaClO_4_ (50/50 *w/w*) solvent. pM values for Ln^3+^–EDTA systems are used for broad comparison purposes since EDTA is one of the most popular and powerful chelating agents.

This procedure is implemented in Figure 6, which presents pM values calculated for Ln^3+^–harzianic acid systems using the pertinent equilibrium constants determined in this paper in the CH_3_OH/0.1 M NaClO_4_ (50/50 *w/w*) solvent and, for broad comparison purposes, pM values calculated for Ln^3+^–EDTA systems, using equilibrium constants in water taken from the literature [45]. Figure 6 suggests that among the investigated Ln^3+^ cations, harzianic acid most efficiently complexes La^3+^ (pLa = 17.44). In fact, according to the pM criterion, lanthanum appears to be more efficiently complexed by harzianic acid in the CH_3_OH/0.1 M NaClO_4_ (50/50 *w/w*) solvent than by EDTA in water at pH = 7.4. Furthermore, harzianic acid displayed a significantly decreased binding ability toward samarium because pSm = 13.65 is the lowest pM value observed in Figure 6 for Ln^3+^–harzianic acid systems. 

### 2.3. Ecotoxicity Tests on Raphidocelis subcapitata, Daphnia magna, and Aliivibrio fischeri

Exposure of organisms and microorganisms to rare-earth elements may have adverse effects, and these elements are emerging contaminants of soil and surface and groundwaters [46,47]. 

Among the rare-earth elements investigated in this work, gadolinium is the one that poses the main concern because of its widespread use in biomedical applications [36]. For this reason, we selected gadolinium as a placeholder for rare-earth elements, to establish the effect of harzianic acid on gadolinium biotoxicity in ecotoxicological assays consisting of aquatic biotoxicity tests performed against selected organisms: *A. fischeri*, *R. subcapitata,* and *D. magna*.

In order to determine the median effective concentration (EC50) and the effective concentration at 10% effect (EC10) of harzianic acid and gadolinium, ecotoxicological tests were performed at different concentrations of harzianic acid (3.5-1.75-1.4-0.9-0.7-0.4 µM) and gadolinium (8.4-4.2-3.3-1.6-0.8-0.4 µM). Results of these bioassays are reported in Table 4. 

**Figure 7:**
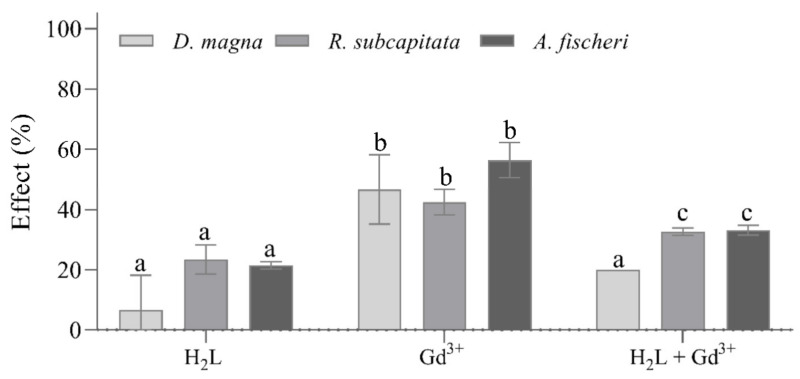
Results of ecotoxicity tests performed on *A. fischeri, R. subcapitata,* and *D. magna* exposed to 0.7 µM harzianic acid, to 3.7 µM gadolinium and to 3.7 µM Gd^3+^ + 0.7 µM harzianic acid. Data are reported as mean ± SD (*n* = 3). Letters (a–c) indicate significant differences between treatments; the level of significance was set at α = 0.05 (ANOVA). H_2_L = harzianic acid.

The sensitivity of *A. fischeri* to harzianic acid is lower than that of *R. subcapitata*, which has about the same sensitivity to harzianic acid as *D. magna*. *A. fischeri* is also less sensitive to gadolinium than *D. magna* and *R. subcapitata*, but the sensitivity to gadolinium of *D. magna* (EC50 = 1.1 µM) appears to be somewhat greater than that of *R. subcapitata* (EC50 = 1.2 µM). The EC50 value of gadolinium for *R. subcapitata* measured here (3.5 µM) compares well with the one assessed by Joonas et al. [36], who reported an EC50 value of 4.1 µM. The EC50 values for *D. magna* and *A. fischeri* determined in this paper are somewhat lower than EC50s obtained by González et al. [48]. This difference could be due to the gadolinium source, that is Gd(NO_3_)_3_ instead of GdCl_3_. EC50 and EC10 values of harzianic acid could not be compared with previous studies because this is the first study in which the ecotoxicological effects of harzianic acid were investigated. For the results shown in Table 4, ecotoxicity tests were performed by exposing *A. fischeri*, *R. subcapitata*, and *D. magna* to 0.7 µM harzianic acid, to 3.7 µM gadolinium, and to 0.7 µM harzianic acid + 3.7 µM gadolinium. Results reported in Figure 7 indicate that the toxicity of gadolinium is reduced when harzianic acid is present, presumably because of the formation of Gd^3+^–harzianic acid complexes. The residual toxicity (33%, 32%, and 20% for *A. fischeri*, *R. subcapitata*, and *D. magna*, respectively) observed after exposition to (0.7 µM harzianic acid + 3.7 µM gadolinium) is expected because harzianic acid concentration is stoichiometrically insufficient for completely converting gadolinium into Gd^3+^–harzianic acid complex species.

## 3. Materials and Methods

### 3.1. Reagents and Their Analysis

Stock solutions of tripositive rare-earth cations were prepared by dissolving their high-purity oxides in concentrated hydrochloric or perchloric acids (Merck, Darmstadt, Germany). The exact concentration of each metal solution was determined as described by Kolthoff et al. [49].

The harzianic acid used in this study was extracted from cultures of an L1 strain of *Trichoderma pleuroticola* recovered from a specimen of the mollusk *Melarhaphe neritoides* (Gastropoda, Littorinidae) collected along the coastline of the isle of Procida, Bay of Naples, Italy [16]. Mycelial plugs from actively growing cultures were inoculated in 1 L Erlenmeyer flasks containing 500 mL of potato dextrose broth (PDB, Himedia, Einhausen, Germany). Cultures were kept in darkness at 25 °C for 3 weeks and, after the incubation period, were filtered through a 0.45 µm filter. The culture filtrate of *T. pleuroticola* was exhaustively extracted in acid conditions with ethyl acetate (EtOAc). The crude extract was dissolved in chloroform and extracted with a saturated solution of NaHCO_3_. The aqueous phase was acidified and extracted with EtOAc to obtain a residue identified as harzianic acid comparing the NMR data with previous reports [39]. 

### 3.2. HPLC–MS Q-TOF Analyses

Solutions were prepared by mixing 500 µL of 2 mM solution of each Ln^3+^ cation (La^3+^, Nd^3+^, Sm^3+^, Gd^3+^) and 500 µL of harzianic acid 1 mg mL^−1^ in methanol. Then, 7 µL of each sample were injected in an Agilent high-performance liquid chromatography (HPLC) 1260 Infinity Series (Agilent Technologies, Santa Clara, CA, USA) coupled with a quadrupole-time-of-flight (Q-TOF) mass spectrometer model G6540B (Agilent Technologies) with a Dual ESI source (Agilent Technologies). The injected volumes were eluted with 0.1% formic acid in acetonitrile at a flow rate of 0.3 mL·min^−1^ to the mass spectrometer. The system operated in positive ion mode and a standard solution of purine and hexakis(1H,1H,3H-tetrafluoropentoxy)phosphazene was infused to obtain the real-time lock mass correction. All parameters and acquisitions were set using the Agilent MassHunter Data Acquisition Software, rev. B.05.01 (Agilent Technologies).

### 3.3. Preparation of Test Solutions for CD and UV–Vis Spectrophotometric Measurements

Acquisition workflow for CD and UV-Vis measurements on solutions of rare-earth cations and harzianic acid contemplates the acquisition, for each element, of two sets of CD and UV-Vis spectra on solutions of accurately known analytical composition (specified by the four analytical variables $C_{\mathrm{Ln}}$ M, $C_{H_{2}L}$ M, $C_{H}$ M, and $C_{\mathrm{OH}}^{\;}$ M) and pH (= −log[H^+^]). In fact, to help in the interpretation of spectra, solutions in each set were planned to have the same ligand-to-metal ratio (i.e., $C_{H_{2}L}/C_{\mathrm{Ln}}$= *constant*), respectively, very close to 1 for the first set and very close to 2 for the second set (Figure 3). To reach this goal, we employed four stock solutions, which, by analysis or preparation, contained accurately known concentrations of Ln(ClO_4_)_3_ or Ln(Cl)_3_ ($C_{\mathrm{Ln}}^{0}$ M), harzianic acid ($C_{H_{2}L}^{\;0}\;M$), HClO_4_ ($C_{H}^{0}\;M$), and NaOH ($C_{\mathrm{OH}}^{0}\;M$) in the CH_3_OH/0.1 M NaClO_4_ (50/50 *w*/*w*) solvent; and a potentiometric apparatus constituted by a multi-neck titration vessel equipped with a Metrohm AG (Herisau, Switzerland) 60102-100 pH sensitive glass electrode (GE) and an Ag/AgCl_(s)_/0.1 M NaCl/(0.1 M NaClO_4_/CH_3_OH, 50/50 *w/w*) double junction reference electrode (RE).

The experiment started by introducing a fixed volume, $V_{H}$ mL, of the HClO_4_ stock solution in the titration vessel, which was kept in an air thermostat at 25 ± 0.1 °C. This realized a potentiometric cell, GE/Solution/RE, whose potential, $E_{G}\;\mathrm{Volt}$, under the present conditions, can be expressed by the following simple relation (4):

(4) $$E_{G}\left(\mathrm{Volt}\right)=E_{G}^{0}\left(\mathrm{Volt}\right)+Slope\cdot \log\left[H^{+}\right]\;\;$$

In order to evaluate the constants $E_{G}^{0}$ and *Slope* in Equation (4), the solution in the potentiometric vessel was alkalimetrically titrated by stepwise addition of accurately measured volumes of the $C_{\mathrm{OH}}^{0}$ M stock solution of NaOH. In all experiments, the alkalimetric titration ended when the same total volume, $V_{\mathrm{OH}}$, of NaOH solution had been added, and the solution in the potentiometric vessel had attained a fixed volume equal to $\left(V_{H}+V_{\mathrm{OH}}\right)$ mL. After the alkalimetric titration, accurately measured volumes of the $C_{\mathrm{Ln}}^{0}$ M solution of Ln^3+^ and of the $C_{H_{2}L}^{0}$ M solution of harzianic acid were added to the $\left(V_{H}+V_{\mathrm{OH}}\right)$ mL of solution in the titration vessel. The added volumes of harzianic acid and metal solutions determined the ligand-to-metal ratio in the resulting solution. After this, the solution was brought to its final pH by adding a measured volume of the $C_{\mathrm{OH}}^{0}$ M solution of NaOH. The volume of NaOH solution added in this step determined the values of $C_{\mathrm{Ln}}$, $C_{H_{2}L}$ and pH in the final solution, which would be used for analysis with the CD and UV–Vis spectrometers but, obviously, did not change the ligand-to-metal ratio. Subsequently, sufficient time was allowed for chemical equilibrium to be established and for the glass electrode potential, $E_{G}$, to achieve a constant value, which persisted for at least 15 min within ±0.1 mV. Thus, the free proton concentration of the solution in the titration vessel was readily calculated from Equation (5), and the measured $E_{G}$ as follows:

(5) $$E_{G}=E_{G}^{0}+Slope\cdot \log\left[H^{+}\right]\to \;\mathrm{pH}=-\log\left[H^{+}\right]=\frac{E_{G}^{0}-E_{G}}{Slope}\;\;\;\;\;\;\;\;\;\;\;\;\;\;\;\;\;\;\;\;\;\;\;\;\;\;\;\;$$

Finally, appropriate volumes of solution were withdrawn from the titration vessel and submitted for CD and UV measurements at 25.0 °C. By this procedure, using fixed volumes of the $C_{\mathrm{Ln}}^{0}$ M stock solution of Ln^3+^ and the $C_{H_{2}L}^{0}$ M stock solution of harzianic acid for each set of CD and UV–Vis measurements, the ratio $C_{H_{2}L}/C_{\mathrm{Ln}}$ was kept the same in each dataset for each element (e.g., for Gd^3+^, $C_{H_{2}L}/C_{\mathrm{Ln}}$ = 1.004 for one dataset and $C_{H_{2}L}/C_{\mathrm{Ln}}$ = 1.991 for the other). Values of $C_{\mathrm{Ln}}$ ranged broadly between ≈9 × 10^−5^ M and ≈4 × 10^−5^ M, while values of $C_{H_{2}L}$ ranged between ≈1.8 × 10^−4^ M and ≈9 × 10^−5^ M.

UV–Vis spectra were recorded by Cary model 5000 Spectrophotometer by Varian C. (Palo Alto, CA, USA), from 200 to 600 nm (optical path 1 or 0.2 cm) at 25.0 °C, under a constant flow of nitrogen.

The far UV–CD spectra were recorded with a JASCO CD spectrometer model J-715 (Tokyo, Japan), from 250 to 500 nm (optical path 1 or 0.2 cm) at 25.0 °C, under a constant flow of nitrogen.

### 3.4. Ecotoxicity Tests

Toxicity tests with *A. fischeri, R. subcapitata,* and *D. magna* were carried out on solutions of different concentrations of Gd and harzianic acid prepared in ISO medium [50]. Concerning the toxicity tests on exposure to Gd^3+^ + harzianic acid, a test solution containing GdCl_3_ (3.7 µM) and harzianic acid (0.7 µM) was prepared in ISO medium [50]. According to quality assurance and quality control procedures, ecotoxicity tests included the assessment of negative and positive controls according to the corresponding standard method. In particular, a bioluminescence inhibition assay was carried out using *A. fischeri* (NRRL-B-11177) according to ISO 11348 [51]. The luminescence was measured with a Microtox® analyzer (Model 500, AZUR Environmental) after 30 min at 15 °C. Tests were carried out in triplicate. Data were analyzed with Microtox Omni software, and results are expressed as a percentage of bioluminescence inhibition.

The growth inhibition test with *R. subcapitata* was carried out according to ISO 8692 [52]. The initial inoculum contained 10^4^ cell mL^−1^. The specific growth inhibition rate was calculated considering 3 replicates exposed at 20 ± 1 °C for 72 h under continuous illumination (6000 l×). Effect data are expressed as a percentage of growth inhibition.

Newborn daphnids (<24 h old) were exposed to the substrate according to the ISO 6341 [50]. Toxicity tests included 5 daphnids per four replicates in 10 mL of solutions. After 24 h, immobilized organisms were recorded. Whenever possible, toxicity was expressed as median effective concentration (EC50, %) or a percentage of effect at the highest tested concentration (%, *w/v*). The significance of differences between average values of different experimental treatments was assessed by the analysis of variance (ANOVA) considering a significance threshold level always set at 5%. When ANOVA revealed significant differences among treatments, post hoc tests were carried out with Tukey’s test. Statistical analyses were performed using Microsoft® Excel 2013/XLSTAT©-Pro (Version 7.2, 2003, Addinsoft, Inc., Brooklyn, NY, USA).

## 4. Conclusions

Mass spectrometric, circular dichroism, UV–Vis spectrophotometric, and pH measurements were combined to shed light on the interactions of the fungal metabolite harzianic acid with selected rare-earth (i.e., lanthanum, neodymium, samarium, gadolinium) tripositive cations in a CH_3_OH/0.1 M NaClO_4_ (50/50 *w*/*w*) solvent. Harzianic acid can form mono- (LnL^+^) and bis-complexes (LnL_2_^−^) with the considered rare-earth cations. As expected, the formation constants of LnL^+^ complexes (*β*_11_) increased as the ionic radius decreased, but an opposite trend was observed for the stepwise formation constants *K*_12_ = (*β*_12_/*β*_11_), which increased with ionic radius. Due to this trend, lanthanum had the highest value of the *K*_12_ (=11.2) stepwise formation constant and the lowest value of the first formation constant *β*_11_ (=6.3). Thus, the LaL^+^ complex is always a minor species, and the bis-complex, LaL_2_^−^, seems to be formed in a single step directly from La^3+^ through the reaction La^3+^ + 2L^2^^−^ ⇌ LaL_2_^−^ (*β*_12_ = 17.46). Furthermore, among the investigated Ln^3+^ cations, harzianic acid most efficiently complexed La^3+^. In fact, according to the calculated pM values, lanthanum appeared to be more efficiently complexed by harzianic acid in the CH_3_OH/0.1 M NaClO_4_ (50/50 *w/w*) solvent than by EDTA in water at pH = 7.4. 

Finally, ecotoxicological tests suggest that gadolinium biotoxicity decreased with the presence of harzianic acid in the chemical environment, presumably because of the formation of Gd^3+^–harzianic acid complexes.

## Figures and Tables

**Table 1:** Characterization of Ln^3+^/harzianic acid complex by HPLC–ESI–HRMS.

Ion	Observed *m*/*z* of Main Isotopic Peak	Formula	Exact Mass
**Harzianic acid + La(ClO_4_)_3_**			
[H_2_L + H]^+^	366.1921	C_19_H_28_NO_6_	366.1917
[H_2_L + Na]^+^	388.1730	C_19_H_27_NO_6_Na	388.1736
[H_2_L − H + La + ClO_4_]^+^	602.0315	C_19_H_26_NO_6_LaClO_4_	602.0309
[H_2_L + La + 2ClO_4_]^+^	701.9867	C_19_H_27_NO_6_La(ClO_4_)_2_	701.9872
[2H_2_L − 2H + La]^+^	867.2592	C_38_H_52_N_2_O_12_La	867.2584
**Harzianic acid + NdCl_3_**			
[H_2_L + H]^+^	366.1908	C_19_H_28_NO_6_	366.1917
[H_2_L + Na]^+^	388.1728	C_19_H_27_NO_6_Na	388.1736
[2H_2_L − 2H + Nd]^+^	870.2597	C_38_H_52_N_2_O_12_Nd	870.2602
**Harzianic acid + Sm(ClO_4_)_3_**			
[H_2_L + H]^+^	366.1926	C_19_H_28_NO_6_	366.1917
[H_2_L + Na]^+^	388.1741	C_19_H_27_NO_6_Na	388.1736
[2H_2_L − 2H + Sm]^+^	880.2716	C_38_H_52_N_2_O_12_Sm	880.2718
**Harzianic acid + GdCl_3_**			
[H_2_L + H]^+^	366.1921	C_19_H_28_NO_6_	366.1917
[H_2_L + Na]^+^	388.1730	C_19_H_27_NO_6_Na	388.1736
[2H_2_L − 2H + Gd]^+^	886.2765	C_38_H_52_N_2_O_12_Gd	886.2761

**Table 2:** Ratios of harzianic acid to Ln^3+^, concentration of Ln^3+^, and pH employed to acquire spectrophotometric data in Figure 3. The last column relates the reported data to their corresponding curves in Figure 3. O.P. = optical path.

Element	$C_{H_{2}L}/C_{Ln}$	$10^{5}\times C_{Ln}\left(pH\right),\;M$	O.P., cm	Dataset
La	0.995	3.82(2.88); 3.64(3.31); 3.55(4.22); 3.49(7.37); 3.45(7.76); 3.42(8.00); 3.38(8.12)	1	Figure 3A (orange)
	1.992	3.99(2.88); 3.86(3.20); 3.76(3.83); 3.74(4.62); 3.71(6.48); 3.68(7.34)	1	Figure 3A (blue)
Nd	1.000	23.3(2.84); 21.5(4.44); 21.2(6.36); 20.8(7.31); 20.5(7.79); 20.1(8.11); 19.7(8.59)	0.2	Figure 3B (orange)
	1.935	8.26(2.76); 7.78(3.26); 7.60(3.81); 7.54(4.57); 7.46(6.90); 7.42(7.32); 7.37(7.62); 7.04(9.71)	0.2	Figure 3B (blue)
Sm	1.051	3.01(2.91); 2.85(3.76); 2.81(5.59); 2.80(6.30); 2.79(6.81); 2.78(7.29); 2.74(7.86); 2.69(8.24)	1	Figure 3C (orange)
	2.02	8.04(2.94); 7.85(3.17); 7.65(3.71); 7.59(4.12); 7.55 (4.83)	1	Figure 3C (blue)
Gd	1.00	6.44(3.55); 6.36(3.94); 6.32(5.06); 6.26 (6.81); 6.23(7.39)	1	Figure 3D (orange)
	1.99	4.57(3.18); 4.41(4.23); 4.35(7.39); 4.25(9.62)	1	Figure 3D (blue)

**Table 3:** Summary of Ln^3+^–harzianic acid (H_2_L) formation constants. **σ** denotes the estimated standard deviation.

Ln^3+^	Reaction	log (Formation Constant), log *β_pq_* ± *σ*
La^3+^	La^3+^ + L^2^^−^ ⇌ LaL^+^ La^3+^ + 2 L^2^^−^ ⇌ LaL_2_^−^	6.26 ± 0.04 17.46 ± 0.04
Nd^3+^	Nd^3+^ + L^2^^−^ ⇌ NdL^+^ Nd^3+^ + 2 L^2^^−^ ⇌ NdL_2_^−^	7.89 ± 0.04 15.48 ± 0.04
Sm^3+^	Sm^3+^ + L^2^^−^ ⇌ SmL^+^ Sm^3+^ + 2 L^2^^−^ ⇌ SmL_2_^−^	8.07 ± 0.02 13.66 ± 0.03
Gd^3+^	Gd^3+^ + L^2^^−^ ⇌ GdL^+^ Gd^3+^ + 2 L^2^^−^ ⇌ GdL_2_^−^	9.15 ± 0.07 14.14 ± 0.07

**Table 4:** Effective concentration at 10% (EC10) and median effective concentration (EC50) of gadolinium and harzianic acid for *A. fischeri*, *R. subcapitata,* and *D. magna*; EC values are provided as averages (*n* = 3), and values in brackets represent ± 95% confidence limits.

Organism	Compound	EC10 (µM)	EC50 (µM)
*A. fischeri*	Harzianic acid	>3.5	>3.5
	Gadolinium	1.5 (0.7–2.6)	2.6 (1.4–4.0)
*R. subcapitata*	Harzianic acid	0.7 (0.4–0.9)	1.2 (0.8–1.9)
	Gadolinium	0.0 (0.0–0.1)	3.5 (1.4–8.3)
*D. magna*	Harzianic acid	0.7 (0.3–1.1)	1.2 (0.7–2.2)
	Gadolinium	0.0 (0.0–1.3)	1.1 (0.0–14)

## Data Availability

The data that support the findings of this study are available from the corresponding author upon reasonable request.

## Supplementary Materials

The following supporting information can be downloaded at: https://www.mdpi.com/article/10.3390/molecules27061959/s1, Figure S1: Far-UV circular dichroism (CD) spectra of harzianic acid at different pH values, Figures S2–S5: Far-UV circular dichroism (CD) spectra of complexes of harzianic acid and Ln^3+^ at different pH values.

## Sample Availability

Samples of the compound harzianic acid are available from the authors.
