# Supplementary material for: Interaction of the Fungal Metabolite Harzianic Acid with Rare-Earth Cations (La3+, Nd3+, Sm3+, Gd3+)
Source: Molecules. 2022 Mar 17;27(6):1959. doi: 10.3390/molecules27061959 (PMC8954165; doi:10.3390/molecules27061959)
Supplement: Supplementary file 1 [file molecules-27-01959-s001.zip › molecules-1632949-supplementary.pdf]

Supplementary Materials:

Interaction of the Fungal Metabolite Harzianic Acid with Rare-Earth Cations (La^{3+}, Nd^{3+}, Sm^{3+}, Gd^{3+})

Gaetano De Tommaso [1,#], Maria Michela Salvatore [1,2,#], Antonietta Siciliano [3], Alessia Staropoli [2,4], Francesco Vinale [2,5,6], Rosario Nicoletti [4,7], Marina DellaGreca [1], Marco Guida [3], Francesco Salvatore [1,*], Mauro Iuliano [1,*], Anna Andolfi [1,6,*]

[1] Department of Chemical Sciences, University of Naples Federico II, Naples 80126, Italy; gaetano.detommaso@unina.it (G.D.T.), mariamichela.salvatore@unina.it (M.M.S.), dellagre@unina.it (M.DG)
[2] Institute for Sustainable Plant Protection, National Research Council, Portici 80055 (NA), Italy;
[3] Department of Biology, University of Naples Federico II, Naples, Italy, antonietta.siciliano@unina.it (A.S.), marco.guida@unina.it (M.G.)
[4] Department of Agricultural Sciences, University of Naples Federico II, Portici 80055 (NA), Italy; alessia.staropoli@unina.it (A.S.)
[5] Department of Veterinary Medicine and Animal Productions, University of Naples Federico II, 80137Naples, Italy; frvinale@unina.it (F.V.)
[6] BAT Center - Interuniversity Center for Studies on Bioinspired Agro-Environmental Technology, University of Naples Federico II, Portici (NA) 80055, Italy
[7] Council for Agricultural Research and Economics, Research Centre for Olive, Fruit and Citrus Crops, 81100 Caserta, Italy; rosario.nicoletti@crea.gov.it (R.N.)

* Correspondence: frsalvat@unina.it (F.S), mauro.iuliano@unina.it (M.I.); andolfi@unina.it (A.A.); Tel.: +39-081-2539179 (A.A.)
\# These authors contributed equally to this work.

Figure S1. Far-UV circular dichroism (CD) spectra (optical path 0.2 cm) of harziaic acid in CH_3OH/0.1 M $NaClO_4$ (50:50 *w*/*w*) at different pH values: $C_{H_2L} \leq 1.72 \times 10^{-4}$M.

Figure S2. Far-UV circular dichroism (CD) spectra (optical path 1 cm) of $La(ClO_4)_3$ in CH_3OH/0.1 M $NaClO_4$ (50:50 *w*/*w*) at different pH values: (**A**) $\frac{C_{H_2L}}{C_{La}} = 0.995$, $C_{La} \leq 3.82 \times 10^{-5}$M; (**B**) $\frac{C_{H_2L}}{C_{La}} = 1.992$, $C_{La} \leq 3.99 \times 10^{-5}$M.

Figure S3. Far-UV circular dichroism (CD) spectra (optical path 0.2 cm) of $NdCl_3$ in CH_3OH/0.1 M $NaClO_4$ (50:50 *w*/*w*) at different pH values: (**A**) $\frac{C_{H_2L}}{C_{Nd}} = 1.000$, $C_{Nd} \leq 23.3 \times 10^{-4}$M; (**B**) $\frac{C_{H_2L}}{C_{Nd}} = 1.935$, $C_{Nd} \leq 8.26 \times 10^{-5}$M.

Figure S4. Far-UV circular dichroism (CD) spectra (optical path 1 cm) of $Sm(ClO_4)_3$ in CH_3OH/0.1 M $NaClO_4$ (50:50 *w*/*w*) at different pH values: (**A**) $\frac{C_{H_2L}}{C_{Sm}} = 1.051$, $C_{Sm} \leq 8.04 \times 10^{-5}$M ; (**B**) $\frac{C_{H_2L}}{C_{Sm}} = 2.02$, $C_{Sm} \leq 3.01 \times 10^{-5}$M .

Figure S5. Far-UV circular dichroism (CD) spectra (optical path 1 cm) of $GdCl_3$ in CH_3OH/0.1 M $NaClO_4$ (50:50 *w*/*w*) at different pH values: (**A**) $\frac{C_{H_2L}}{C_{Gd}} = 1.00$, $C_{Gd} \leq 6.44 \times 10^{-5}$ M; (**B**) $\frac{C_{H_2L}}{C_{Gd}} = 1.99$, $C_{Gd} \leq 4.57 \times 10^{-5}$M.

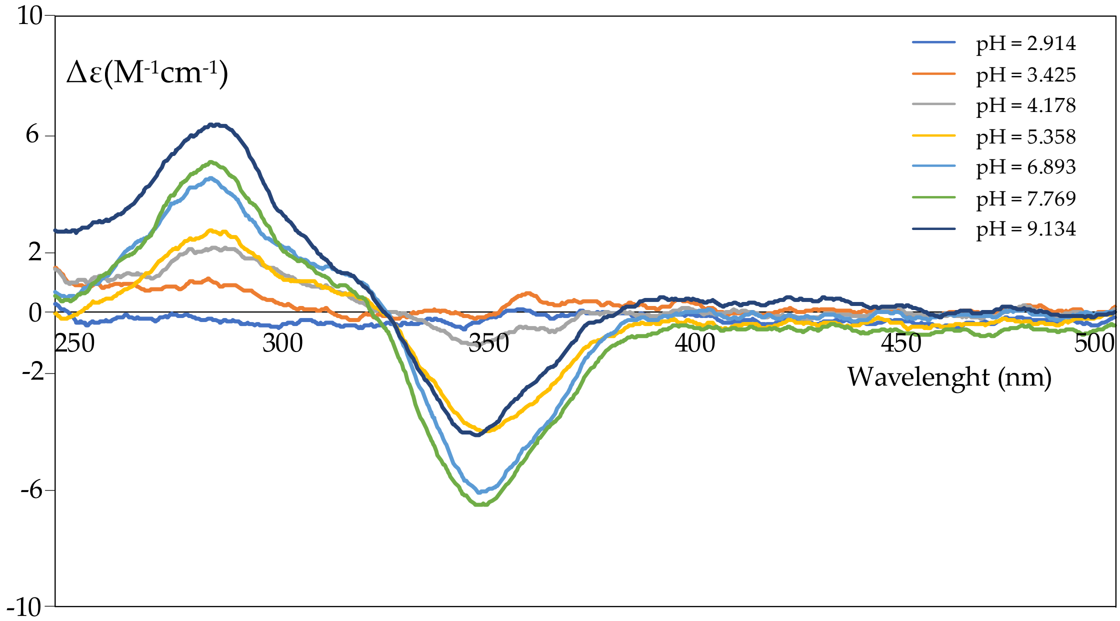

Figure S1. Far-UV circular dichroism (CD) spectra (optical path 0.2 cm) of harziaic acid in CH_3OH/0.1 M $NaClO_4$ (50:50 *w/w*) at different pH values: $C_{H_2L} \leq 1.72 \times 10^{-4}$M.

(A)

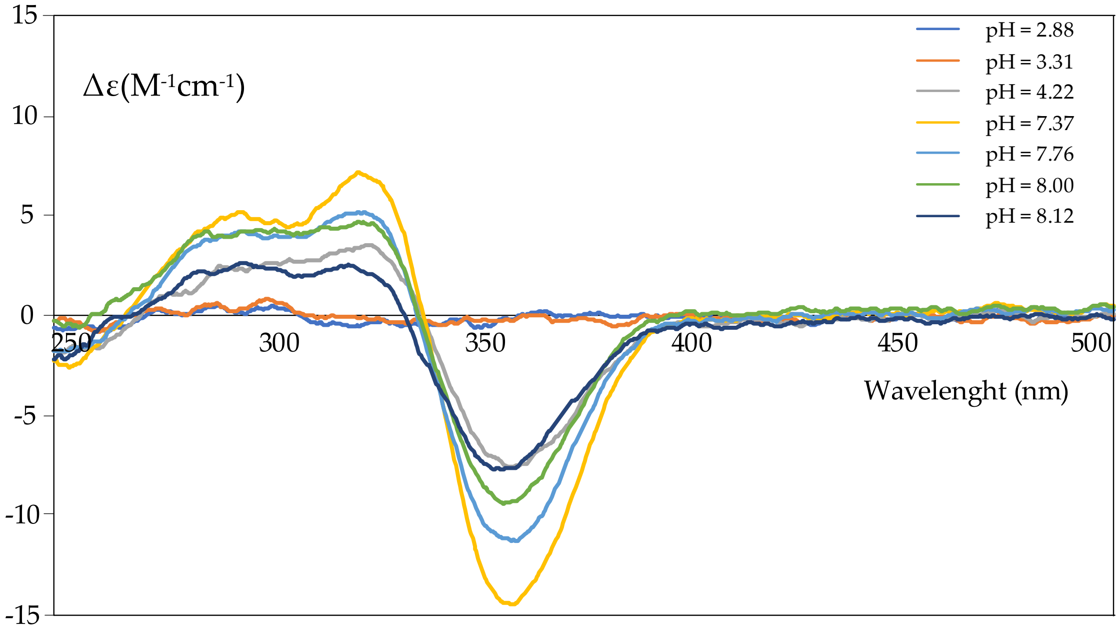

(B)

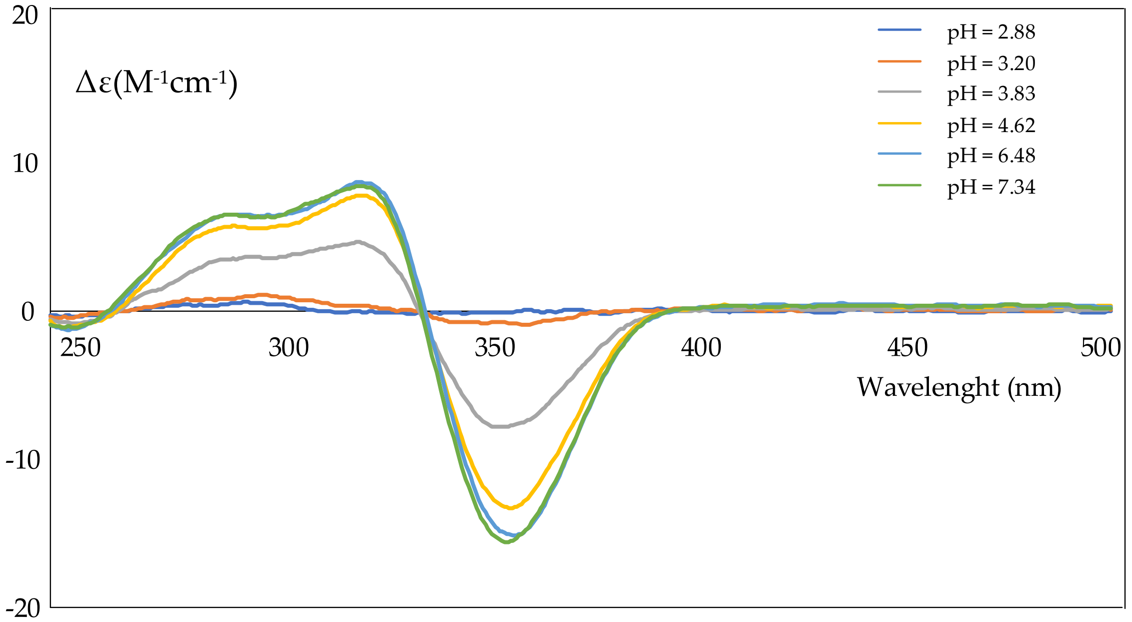

Figure S2. Far-UV circular dichroism (CD) spectra (optical path 1 cm) of $La(ClO_4)_3$ in CH_3OH/0.1 M $NaClO_4$ (50:50 *w*/*w*) at different pH values: (**A**) $\frac{C_{H_2L}}{C_{La}} = 0.995$, $C_{La} \leq 3.82 \times 10^{-5}$M; (**B**) $\frac{C_{H_2L}}{C_{La}} = 1.992$, $C_{La} \leq 3.99 \times 10^{-5}$M.

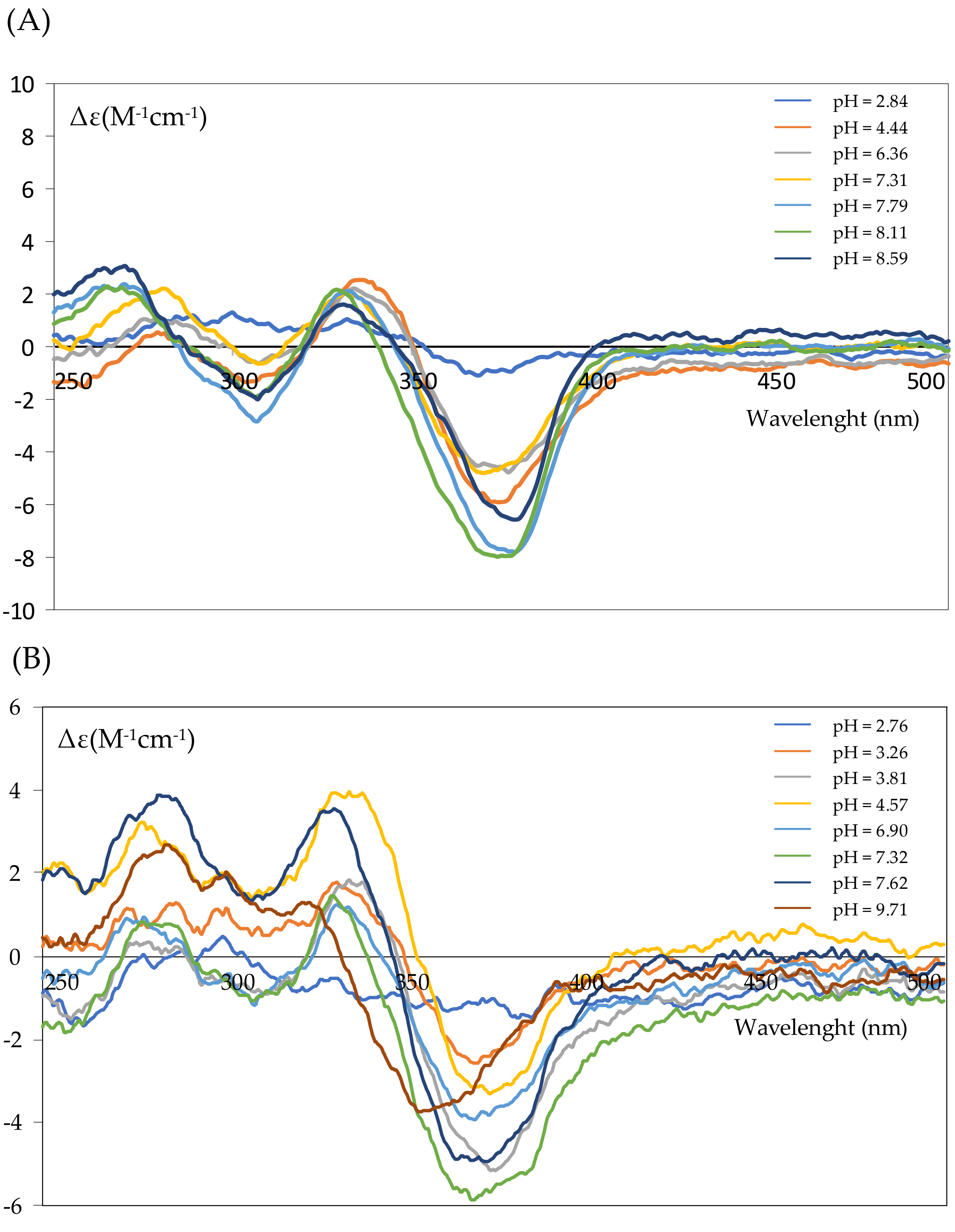

Figure S3. Far-UV circular dichroism (CD) spectra (optical path 0.2 cm) of $NdCl_3$ in CH_3OH/0.1 M $NaClO_4$ (50:50 *w*/*w*) at different pH values: (**A**) $\frac{C_{H_2L}}{C_{Nd}} = 1.000$, $C_{Nd} \le 23.3 \times 10^{-4}$M; (**B**) $\frac{C_{H_2L}}{C_{Nd}} = 1.935$, $C_{Nd} \le 8.26 \times 10^{-5}$M.

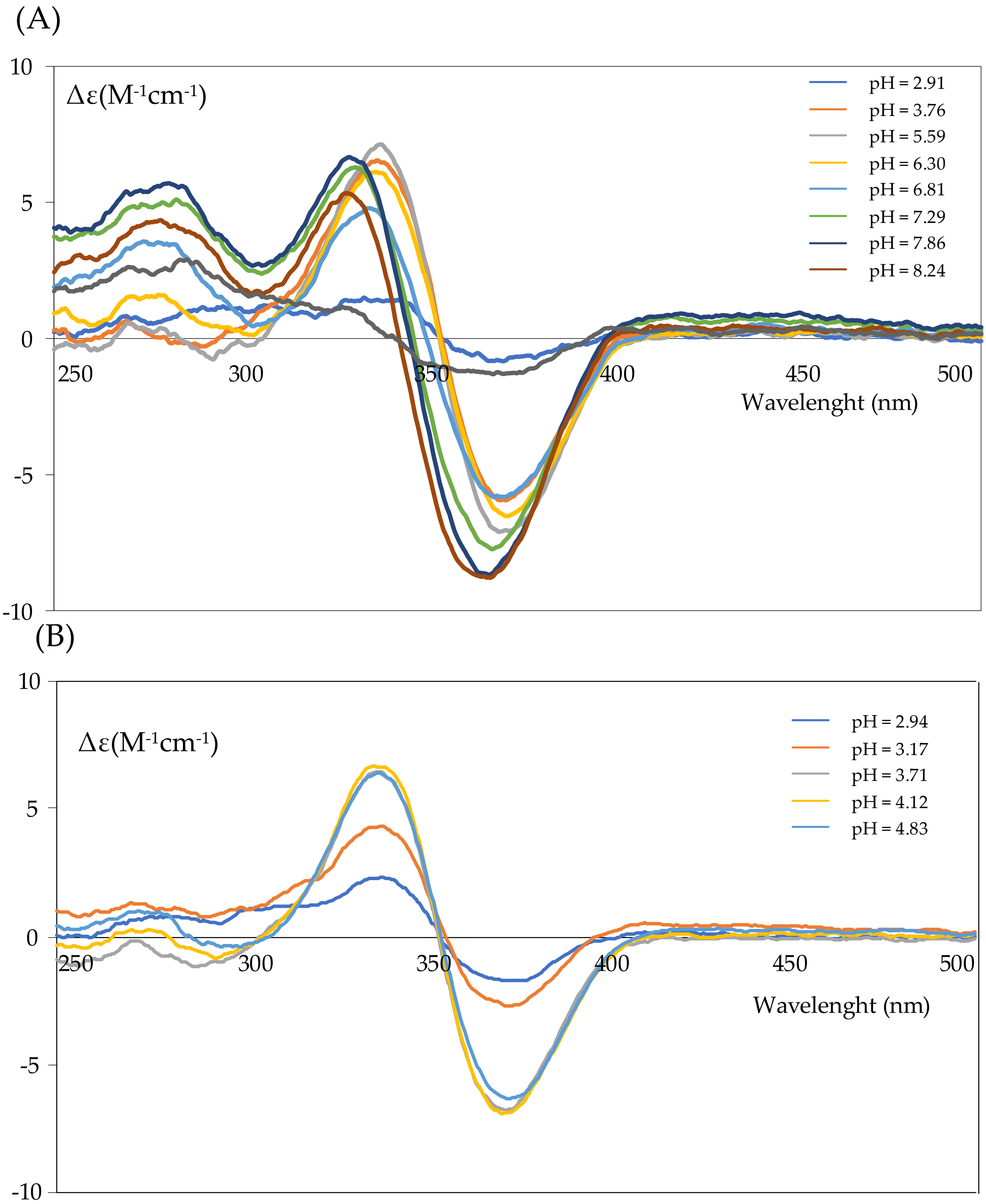

Figure S4. Far-UV circular dichroism (CD) spectra (optical path 1 cm) of $Sm(ClO_4)_3$ in CH_3OH/0.1 M $NaClO_4$ (50:50 *w*/*w*) at different pH values: (**A**) $\frac{C_{\mathrm{H_2L}}}{C_{\mathrm{Sm}}} = 1.051$, $C_{\mathrm{Sm}} \leq 8.04 \times 10^{-5}$M ; (**B**) $\frac{C_{\mathrm{H_2L}}}{C_{\mathrm{Sm}}} = 2.02$, $C_{\mathrm{Sm}} \leq 3.01 \times 10^{-5}$M .

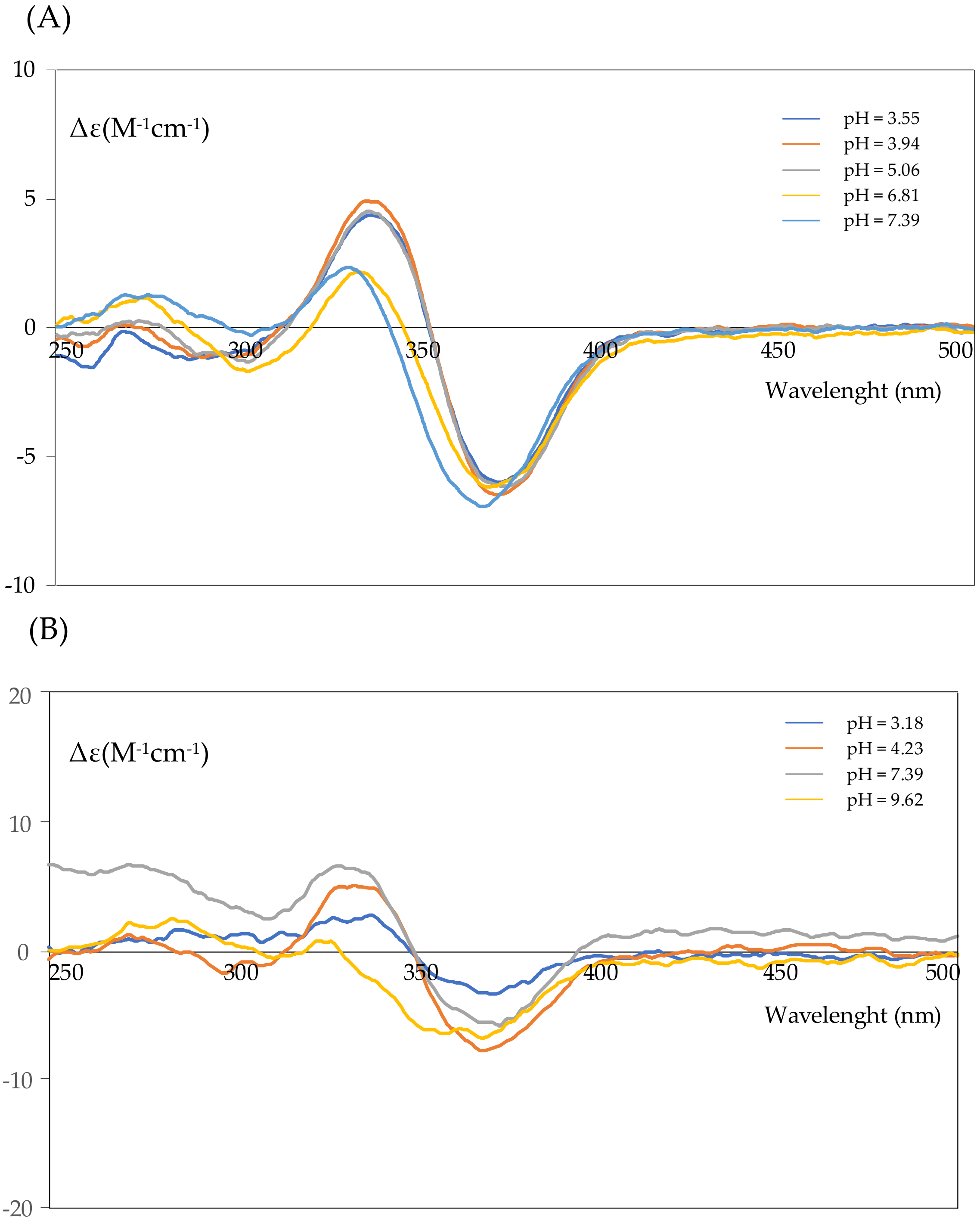

Figure S5. Far-UV circular dichroism (CD) spectra (optical path 1 cm) of $GdCl_3$ in CH_3OH/0.1 M $NaClO_4$ (50:50 *w/w*) at different pH values: (**A**) $\frac{C_{H_2L}}{C_{Gd}} = 1.00$, $C_{Gd} \leq 6.44 \times 10^{-5}$ M; (**B**) $\frac{C_{H_2L}}{C_{Gd}} = 1.99$, $C_{Gd} \leq 4.57 \times 10^{-5}$M.
